# A personalized iodine delivery rate-based injection protocol in coronary angiography on photon-counting CT

**DOI:** 10.1007/s00330-026-12413-x

**Published:** 2026-03-06

**Authors:** Tim Busselot, Pierpaolo Giordano, Vincent Sneyers, Walter Coudyzer, Kwinten Torfs, Tom Adriaenssens, Hilde Bosmans, Steven Dymarkowski

**Affiliations:** 1 Catholic University of Leuven, Leuven, Belgium; 2 University Hospitals Leuven, Leuven, Belgium

**Keywords:** Computed tomography angiography, Contrast media, Coronary artery disease, Coronary vessels

## Abstract

**Objectives:**

To propose and validate a personalized iodine delivery rate (IDR) based injection protocol for coronary CT angiography (cCTA) on photon-counting CT.

**Materials and methods:**

First, ideal IDR (IDR_IDEAL_) was retrospectively calculated for a HU target of 500 in 55 keV image reconstructions. Next, linear regression analysis was performed with IDR_IDEAL_ and demographic parameters to derive a candidate IDR formula. This was implemented in two validation groups characterized by injection rate (3.5 and 5.0 mL/s). Here, coronary enhancement was quantified and equivalence assessed for a predefined HU range (500 ± 50). Additionally, a reader study assessed perceived enhancement of the coronary tree.

**Results:**

This IRB-approved study used a retrospective cohort of 162 patients (group A: 58 ± 12 years; 81 men) and two prospective cohorts of 51 patients (group B1: 60 ± 13 years; 22 men and group B2: 59 ± 12 years; 30 men). IDR_IDEAL_ correlated best with fat-free mass (FFM) (r = 0.67) and was integrated for contrast personalization. Prospectively, mean coronary enhancement was 533 ± 97 HU and 528 ± 68 HU for both groups (*p* = 0.79) with mean IDR values of 1.01 ± 0.11 gI/s and 1.07 ± 0.14 gI/s. However, distribution variances were significantly different (*p* = 0.015). Subjective scoring showed no differences between the two groups on overall and per-vessel level (*p* > 0.05).

**Conclusion:**

A personalized, IDR-based injection protocol for cCTA was proposed and validated. FFM was best for IDR_IDEAL_ prediction. Higher injection rates provided more precise coronary enhancement.

**Key Points:**

***Question**** Virtual mono-energetic images lead to low iodine delivery rate settings, but the impact on coronary enhancement is not clear*.

***Findings**** An iodine delivery rate-based injection protocol implementing personalized contrast volume dilutions with set injection duration and high injection rate improved coronary enhancement*.

***Clinical relevance**** Iodine delivery rate fine-tuning is a promising approach for coronary CT angiography with low iodine volumes. High injection rates provide more accurate and precise coronary enhancement*.

**Graphical Abstract:**

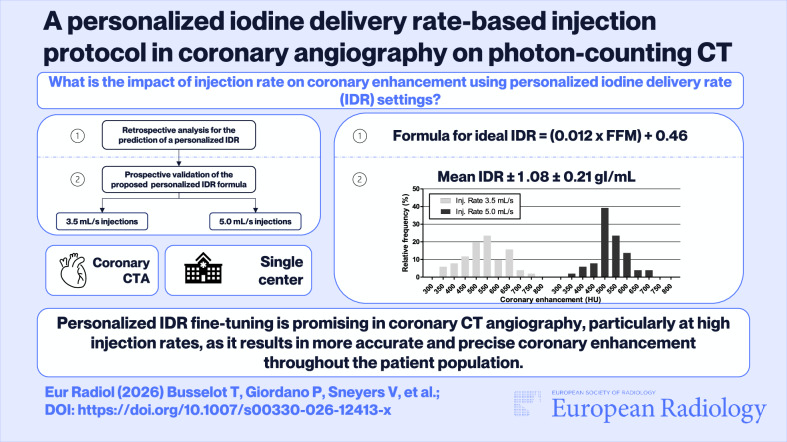

## Introduction

Photon-counting CT (PCCT) is becoming an increasingly important innovation in the field of cardiovascular imaging, promising high-quality cardiac examinations with the possibility to capture spectral information as part of the clinical routine in coronary CT angiography (cCTA) and reconstruct CT images as if they were acquired with mono-energetic beams. Compared to other scanners, there is also no compromise in scan time or temporal resolution, and PCCT is also particularly effective at low dose settings [1, 2]. The opportunity to select virtual mono-energetic images (VMI) allows for the production of images optimized for a specified clinical task [3–6]. In cCTA, optimal energies must be selected for both vessel enhancement and reliable quantification of atherosclerotic plaques.

The injection of iodinated contrast media (ICM) is essential for visualizing the coronary arteries and should ideally lead to an enhancement of 350 to 500 Hounsfield Units (HU) [7, 8]. Contrary to polychromatic images, where vessel enhancement is dependent on set tube voltage [9, 10], dual-energy CT systems allow to use optimized VMI levels [11–13]. The possibility of reducing iodine load using low VMI levels has already been studied for more than 10 years, including cardiovascular imaging [14–17]. With the introduction of PCCT, ICM injection protocols should be reinvestigated, and more in particular if a set HU range is aimed for in the entire patient cohort.

Personalized ICM injection protocols should lead to appropriate vessel enhancement, while keeping the total iodine dose (TID) minimal. Previous studies have already demonstrated the association of various body habitus metrics with vessel enhancement in cCTA scans [9, 18–29]. However, in addition to body habitus, ICM characteristics and injection protocol settings cannot be ignored. In practice, selecting optimal injection protocol parameters is a multi-parameter challenge [10, 26].

The present study focused on iodine delivery rate (IDR), which represents the amount of iodine injected per second. Several publications have already shown the utility of IDR to target appropriate vascular enhancement in clinical practice: using a set injection time, a fixed IDR can be reached by adjusting the injection rate and iodine volume [30–34]. However, the impact of varying injection rate on coronary enhancement in cCTA is less well documented and could potentially introduce negative implications when too low, provided that VMIs enable to reduce these IDR values considerably [35].

The aim of this study is twofold: (1) to propose a personalized IDR-based contrast formula by using easily obtainable body metrics for cCTA on a PCCT, and (2) to investigate the impact of injection rate on coronary enhancement, implementing personalized contrast volume dilutions in a fixed injection time setting. The ultimate aim is to propose a validated injection protocol to reach a set coronary enhancement throughout the entire patient population.

## Materials and methods

### Study design and patient population

This mono-centric study was evaluated and approved by the local IRB of the University Hospitals Leuven, Belgium (S58042 and S70047). All included patients were referred for a clinically indicated cCTA scan to exclude coronary artery disease (CAD). Clinical indications comprised atypical thoracic pain, dyslipidemia, familial history of CAD and coexistence of multiple risk factors [36]. Inclusion criteria were: age older than 18 years, scanning performed with the high-pitch scan mode using predefined scan and injection timings. Renal dysfunction (eGFR < 30 mL/min/1.73 m^2^), known hypersensitivity to ICM and pregnancy were the predefined exclusion criteria.

**Fig. 1:**
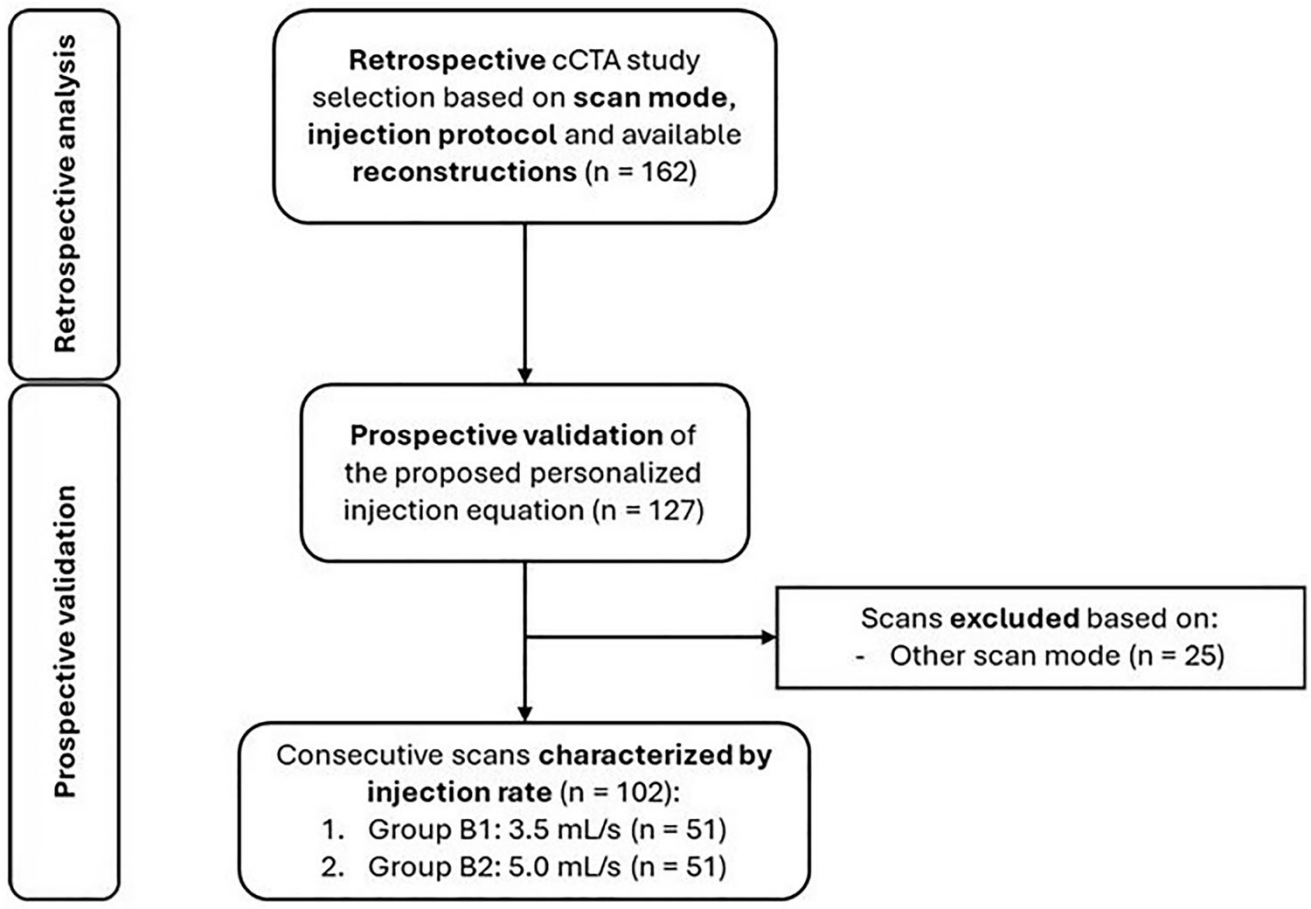
Flowchart of the study. cCTA, coronary CT angiography

The study comprised of (1) a retrospective study (group A) to propose a personalized formula to calculate an injection volume and (2) a prospective study to validate latter formula and to verify the impact of a standardized injection rate, 3.5 and 5.0 mL/s, in two study groups (group B1 and B2 respectively) (Fig. 1). The retrospective cohort comprised of eligible cCTA scans throughout 3 months from May 2024 to July 2024. For the prospective cohort, 127 consecutive patients were enrolled from October 2024 to December 2024, of which 25 patients were excluded because the high-pitch scan mode was not preferable. In both retrospective and prospective study groups, no extravasation events were recorded.

### CT scan acquisition

All scans were performed on a first-generation dual-source PCCT scanner (NAEOTOM Alpha.Peak, Siemens Healthineers) equipped with two cadmium telluride detectors (collimation: 144 × 0.4 mm; gantry rotation speed: 0.25 s). Patients received 5 mg of sublingual nitroglycerin and, if resting heart rate exceeded 70 beats per minute, intravenous beta-blockers were administered prior to scanning. The scan protocol was performed in the electrocardiogram-gated high-pitch spectral acquisition mode (QuantumPlus scan mode, Siemens Healthineers) during one heart cycle, with a tube voltage of 140 kVp, an image quality (IQ) level chosen as a function of the calcium score (limited calcium load, IQ level = 50; else, IQ level = 110) and automated tube current modulation (CareDose4D, Siemens Healthineers) [37]. Contrast timing was defined using the bolus-tracking technique, with a region of interest (ROI) in the descending aorta using a threshold of 140 HU at 90 kVp. A scan delay of 7 s was set after the HU limit was reached.

### Contrast injection protocol

For both study parts, a biphasic injection protocol was performed with a dual-head power injector (Dual Shot alpha 7, Nemoto Kyorindo Co.) (Table 1). A 17 s ICM bolus (Visipaque 320 (iodixanol 320 mgI/mL) and Omnipaque 350 (iohexol 350 mgI/mL)) was injected into, preferably, the antecubital vein through an 18-G intravenous catheter. In the retrospective cohort, this bolus was undiluted and characterized by an injection rate (3.0–4.0 mL/s) and volume (51–68 mL) determined with a simple in-house weight-based rule. In the prospective cohort, this bolus was characterized by a personalized IDR (based on the retrospectively proposed personalized formula (Eq. 10)) and a set injection rate (group B1 = 3.5 mL/s and group B2 = 5.0 mL/s). Next, ICM dilution (ICM:saline) was calculated based on the personalized iodine concentration, derived from the personalized IDR, and the base ICM concentration (Eq. 1). This bolus was followed by a saline chasing bolus of 30 mL at an injection rate matched with the primary bolus.

1 $$\% \,{{{\rm{dilution}}}}=\left(1-\left(\frac{\frac{{{{\rm{Bolus\,Volume}}}}\times {{{\rm{Personalized}}}}\; {{{\rm{Iodine}}}}\; {{{\rm{Concentration}}}}}{{{{\rm{Base}}}}\; {{{\rm{ICM}}}}\; {{{\rm{Concentration}}}}}}{{{{\rm{Bolus}}}}\; {{{\rm{Volume}}}}}\right)\right)\times 100 \%$$

**Table 1:** Injection protocol characteristics

	Retrospective group A	Group B1 Injection rate = 3.5 mL/s	Group B2 Injection rate = 5.0 mL/s
ICM bolus			
IDR selection	Weight-based	FFM_D_-based*	FFM_D_-based*
Injection time (s)	17	17	17
Injection rate (mL/s)	3.0–4.0	3.5	5.0
Injection volume (mL)	51–68	60	85
ICM dilution†	No	Yes, if neededˠ	Yes, if neededˠ
ICM brand˜	Visipaque 320, Omnipaque 350	Omnipaque 350	Omnipaque 350
Base concentration (mgI/mL)	320, 350	350	350
Saline chasing bolus			
Injection rate (mL/s)‡	3.0–4.0	3.5	5.0
Injection volume (mL)	30	30	30

*IDR* iodine delivery rate, *FFM*_*D*_, fat-free mass based on the Deurenberg formula, *ICM* iodinated contrast media

* Using Eq. 10, derived from the retrospective part of this study

ˠ Variable, depending on the predicted personalized ICM concentration and base concentration (Eq. 1)

† ICM bolus was diluted by injector settings, using saline

˜ In the retrospective group, 80% received Visipaque 320 and 20% received Omnipaque 350

‡ Matched with set injection rate of ICM bolus

#### Proposing a formula for ideal IDR prediction using retrospective data

For each scan in the retrospective cohort, the retrospective IDR (IDR_RP_) was calculated using the documented injection rate and iodine concentration (Eq. 2). Next, coronary enhancement was defined as the averaged HU value (HU_RP_) from HU measurements in the ascending aorta (at the level of the ostium of the left main coronary artery) and in the proximal part of the right coronary artery. For these measurements, a circular ROI was drawn in the vessel lumen, obviating the coronary vessel wall. All images were reconstructed in an identical manner, using a Bv44 kernel and slice thickness of 0.4 mm, and enhancement measurements were performed on a VMI level of 55 keV.

2 $${{{\rm{IDR}}}}={{{\rm{ICM}}}}\; {{{\rm{concentration}}}}\times {{{\rm{Injection}}}}\; {{{\rm{Rate}}}}$$

**Fig. 2:**
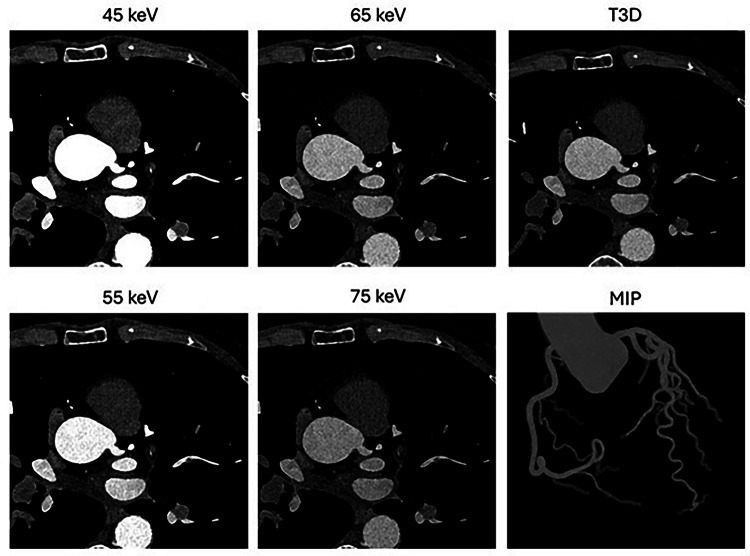
Representative VMI reconstructions (including maximum intensity projection (MIP) at 55 keV) of a cCTA scan from the prospective group, characterized by an injection rate of 5.0 mL/s, which reached the ideal coronary enhancement of 500 HU at 55 keV (WW: 600, WL: 200). Contrast enhancement reduces with increasing keV. cCTA, coronary CT angiography; VMI, virtual mono-energetic image

In this study, a HU value of 500 in the 55 keV VMI reconstructed images was defined as the ideal HU enhancement (HU_IDEAL_). This relatively high target was chosen to guarantee good coronary enhancement when increasing the VMI keV levels, as this could impact stenosis grading [3–5]. However, at this moment of the study, the ideal keV VMI image choice for lumen and plaque quantification was not yet fixed, and therefore the guidelines from the vendor were applied. Figure 2 showcases an example of these different VMI reconstructions in a patient with ideal coronary enhancement.

From IDR_RP_, HU_RP_ and HU_IDEAL_ the ideal IDR (IDR_I__DEAL_) was then calculated for all patients based on the basic principle that IDR and HU enhancement relate linearly in a first approach (Eq. 3) [29].

3 $${{{{\rm{IDR}}}}}_{{{{\rm{IDEAL}}}}}=\frac{{{{{\rm{HU}}}}}_{{{{\rm{IDEAL}}}}}\times {{{{\rm{IDR}}}}}_{{{{\rm{RP}}}}}}{{{{{\rm{HU}}}}}_{{{{\rm{RP}}}}}}$$

Age, gender, and various body metrics were collected and correlated with the calculated IDR_IDEAL_. Based on literature, the following body metrics were selected: length (L), body weight (BW), water equivalent diameter (WED) [38], body mass index (BMI), body surface area (BSA), lean body weight (LBW) and fat-free mass, the latter calculated with the Deurenberg formula (FFM_D_) [39]. Equations 4—9 show these metrics.

4 $${{{\rm{BMI}}}}\left(\frac{{{{\rm{kg}}}}}{{{{{\rm{m}}}}}^{2}}\right)=\frac{{{{\rm{BW}}}}\left({{{\rm{kg}}}}\right)}{{\left({{{\rm{L}}}}\left({{{\rm{m}}}}\right)\right)}^{2}}$$

5 $${{{\rm{BSA}}}}\left({{{{\rm{m}}}}}^{2}\right)=\frac{\surd ({{{\rm{BW}}}}\left({{{\rm{kg}}}}\right)* {{{\rm{L}}}}\left({{{\rm{cm}}}}\right))}{60}$$

6 $${{{\rm{LBW}}}}({{{\rm{kg}}}})=\left(0.3281* {{{\rm{BW}}}}\left({{{\rm{kg}}}}\right)\right)+\left(33.929* {{{\rm{L}}}}\left({{{\rm{m}}}}\right)\right)-29.5336\quad{{{\rm{for}}}}\; {{{\rm{men}}}}$$

7 $${{{\rm{LBW}}}}({{{\rm{kg}}}})=\left(0.29569* {{{\rm{BW}}}}\left({{{\rm{kg}}}}\right)\right)+\left(41.813* {{{\rm{L}}}}\left({{{\rm{m}}}}\right)\right)-43.2933\quad{{{\rm{for}}}}\; {{{\rm{women}}}}$$

8 $${{{{\rm{FFM}}}}}_{{{{\rm{D}}}}}({{{\rm{kg}}}})=	\,{{{\rm{BW}}}}\left({{{\rm{kg}}}}\right)-{{{\rm{BW}}}} \left({{{\rm{kg}}}}\right)\\ 	* \left(\frac{\left(1.2* {{{\rm{BMI}}}}\left(\frac{{{{\rm{kg}}}}}{{{{{\rm{m}}}}}^{2}}\right)\right)+\left(0.23* {{{\rm{Age}}}}\left({{{\rm{y}}}}\right)\right)-16.2}{100}\right)\quad{{{\rm{for\; men}}}}$$

9 $${{{{\rm{FFM}}}}}_{{{{\rm{D}}}}}({{{\rm{kg}}}})=	\,{{{\rm{BW}}}}\left({{{\rm{kg}}}}\right)-{{{\rm{BW}}}}\left({{{\rm{kg}}}}\right)\\ 	* \left(\frac{\left(1.2* {{{\rm{BMI}}}}\left(\frac{{{{\rm{kg}}}}}{{{{{\rm{m}}}}}^{2}}\right)\right)+\left(0.23* {{{\rm{Age}}}}\left({{{\rm{y}}}}\right)\right)-5.4}{100}\right)\quad{{{\rm{for}}}}\; {{{\rm{women}}}}$$

Next, linear regression analysis was performed with the calculated IDR_IDEAL_ and the different patient parameters. The body metric with the highest correlation coefficient was selected as our best candidate for the personalized injection formula, and the resulting regression formula would then be used to predict the IDR_IDEAL_ for routine use in cCTA.

#### Prospective validation of the proposed personalized injection formula

The prospective validation study comprised two groups, characterized by a predefined injection rate (group B1 = 3.5 mL/s and group B2 = 5.0 mL/s). Keeping injection time and scan delay identical to what was used in the retrospective cases, the retrospectively derived candidate formula for IDR_IDEAL_ was used to calculate the dilution rate of the iodine volume, taking into account the predefined injection rate. The resulting coronary enhancement was measured in an identical manner as defined in the retrospective cohort. Additionally, signal-to-noise ratio (SNR) and contrast-to-noise ratio (CNR) were quantified using a circular ROI in the ascending aorta (at the level of the ostium of the left main coronary artery) and in the epicardial adipose tissue [40].

### Subjective quality assessment of coronary artery enhancement

Coronary enhancement of all prospective scans was independently scored by a radiologist (V.S.) with 5 years of experience in cardiovascular radiology and a board-certified radiologist with more than 20 years of experience in cardiovascular imaging (S.D.). Both readers were blinded to the used injection rate and scored all cases from the 55 keV VMI images with identical reconstruction settings. Window width and level (WW:600, WL:200) were set identical for each case, yet no restrictions were applied to adjust these parameters. Coronary enhancement was scored for overall and per-vessel enhancement using a five-point Likert scale characterized by: 5 = excellent, 4 = good, 3 = moderate, 2 = poor and 1 = nondiagnostic.

### Statistical analysis

All statistical analyses were performed using GraphPad Prism (version 5.04, Dotmatics). Descriptive statistics was performed in terms of mean and standard deviation. Normality of the data was assessed with the D’Agostino and Pearson omnibus normality test. Differences between all 3 groups were assessed using an ANOVA or Kruskal–Wallis test, for normal and non-normally distributed datasets, respectively. In the retrospective cohort, correlations were calculated using the Pearson correlation or non-parametric Spearman correlation test, for normal and non-normal datasets, respectively. The correlation coefficients were interpreted using the following categories: 0.00–0.19, very weak; 0.20–0.39, weak; 0.40–0.59, moderate; 0.60–0.79, strong; 0.80–1.00, very strong. Categorical distributions were reported as percentages and evaluated using a Chi-squared test. For the prospective validation cohorts, sample sizes were calculated based on the following null-hypothesis: the difference between the mean HU of the group with personalized ICM injection protocol and the target HU value of 500 HU is outside the equivalence interval. We assumed a power of 0.80, a significance level of 0.05, an anticipated standard deviation of 90 HU and an equivalence limit of 50 HU. The calculated sample size was 51 participants. Equivalence of the measured HU enhancement with the set HU enhancement of 500 ± 50 HU was determined with a two one-sided *t*-test. Differences between both prospective cohorts were assessed using either an unpaired *t*-test or Mann–Whitney test, for normal and non-normally distributed datasets. Variance of coronary enhancement was verified with an F-test. Subjective enhancement scores for both prospective cohorts were compared using a Mann–Whitney test. The significance level throughout the study was set to 0.05.

## Results

### Patient population

In this study, 264 patients were included, subdivided into a retrospective cohort (162 patients) and two prospective cohorts (51 patients each) (Fig. 1). All patient demographics and body metrics are listed in Table 2. There were no significant differences observed in length, weight, age, WED, BMI, BSA, LBW and FFM_D_ among all three groups (*p* > 0.05). Furthermore, there was no significant difference (*p* = 0.44) observed in the CAD-RADS distributions between all three groups.

**Table 2:** Patient demographics and clinical results

	Retrospective group A	Group B1 Injection rate = 3.5 mL/s	Group B2 Injection rate = 5.0 mL/s	*p*-value
No. of patients	162	51	51	
Patient demographics				
Length (cm)	172 ± 10	170 ± 9	172 ± 10	*p* = 0.40
Weight (kg)	78 ± 16	74 ± 14	78 ± 15	*p* = 0.26
Age (years)	58 ± 12	60 ± 13	59 ± 12	*p* = 0.50
WED (cm)	26 ± 3	26 ± 3	26 ± 4	*p* = 0.25
BMI (kg/m^2^)	26 ± 5	25 ± 4	26 ± 4	*p* = 0.42
BSA (m^2^)	1.9 ± 0.2	1.9 ± 0.2	1.9 ± 0.2	*p* = 0.23
LBW (kg)	53 ± 9	51 ± 8	53 ± 9	*p* = 0.23
FFM_D_ (kg)	51 ± 11	48 ± 10	52 ± 11	*p* = 0.14
Gender (% male)	50	43	59	*p* = 0.28
Clinical results*				
CAD-RADS 0 (%)	48	51	43	
CAD-RADS 1 (%)	23	20	27	
CAD-RADS 2 (%)	16	12	20	
CAD-RADS 3 (%)	3	8	8	
CAD-RADS 4A (%)	5	4	2	
CAD-RADS 4B (%)	3	0	0	
CAD-RADS 5 (%)	2	6	0	

All data are summarized as mean ± standard deviation, unless indicated otherwise

*WED* water equivalent diameter, *BMI* body mass index, *BSA* body surface area, *LBW* lean body weight, *FFM*_*D*_ fat-free mass based on the Deurenberg formula, *CAD-RADS* coronary artery disease reporting and data system

* CAD-RADS distributions were tested using a Chi-squared test and showed no significant differences (*p* = 0.44)

### A retrospectively derived cCTA-specific formula to predict the ideal IDR

**Fig. 3:**
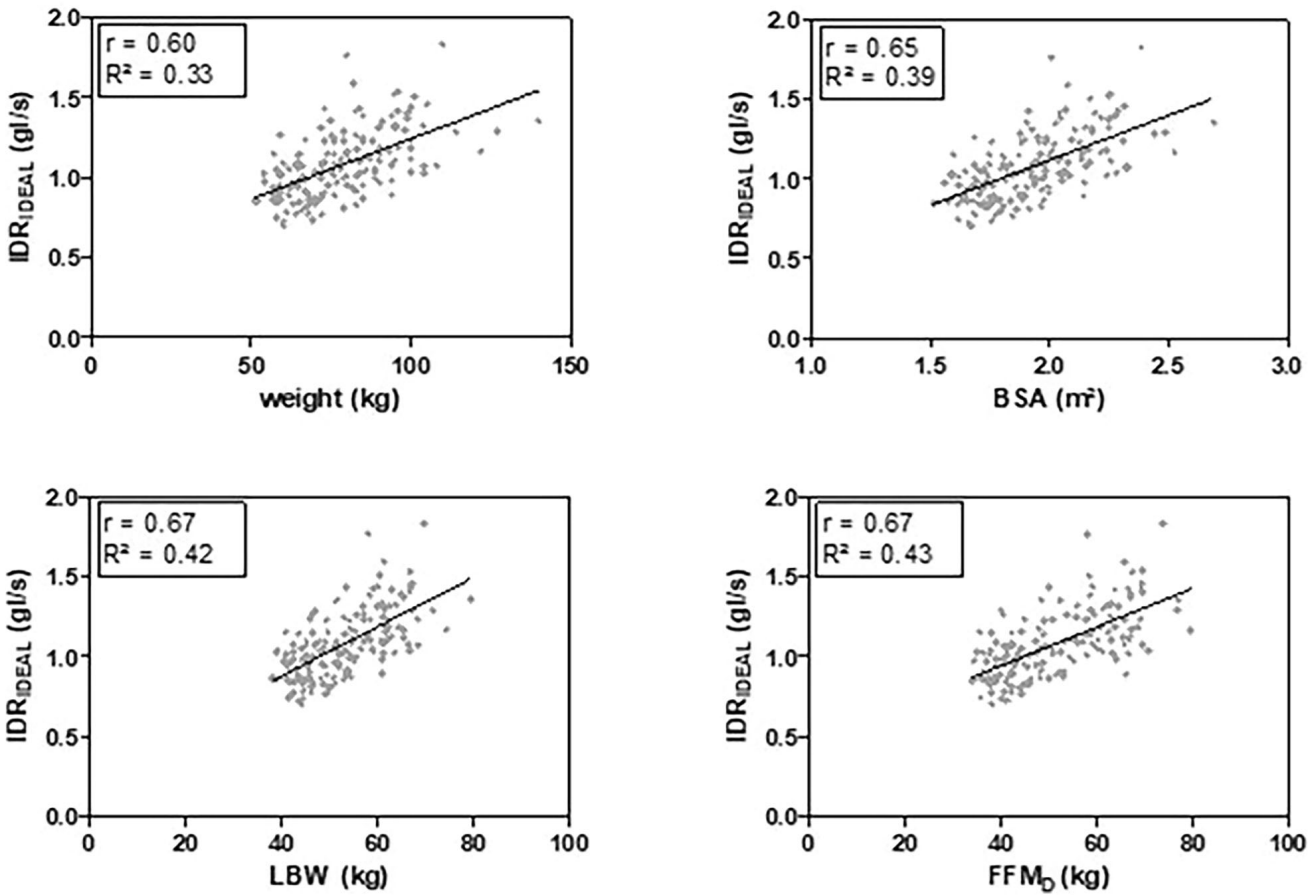
Linear regression analysis of the four highest correlating body metrics IDR_IDEAL_. BSA, body surface area; FFM_D_, fat-free mass based on the Deurenberg formula; IDR, iodine delivery rate; LBW, lean body weight

Coronary enhancement in the retrospective study was 548 ± 90 HU and followed a normal distribution. For each patient, IDR_IDEAL_ was calculated and showed in 59% of patients a possibility for reduction when compared to the IDR_RP_. Only in 22% of cases, the IDR_IDEAL_ showed an increase compared to IDR_RP_. Mean IDR_IDEAL_ was 1.08 ± 0.21 gI/s compared to the observed IDR_RP_ of 1.15 ± 0.13 gI/s. Next, correlation of IDR_IDEAL_ was significant (*p* < 0.05) with all body metrics. Patient age showed a weak negative correlation with IDR_IDEAL_ (r = −0.25). BMI (r = 0.31) and WED (r = 0.38) showed a weak positive correlation, while patient length (r = 0.56) showed a moderate positive correlation. Most importantly, patient weight (r = 0.60), BSA (r = 0.65), LBW (r = 0.67) and FFM_D_ (r = 0.67) showed all strong positive correlations. Linear regressions of the four highest correlating body metrics are shown in Fig. 3. Based on the additional integration of the age parameter in the calculation and slightly better R^2^ value, FFM_D_ was selected over LBW as predictor for IDR_IDEAL_. Therefore, the linear regression of IDR_IDEAL_ as a function of FFM_D_ was adopted to predict IDR_IDEAL_ (Eq. 10).

10 $${\rm IDR}_{\rm IDEAL} = 0.012 \, \times \,{\rm FFM}_{\rm D} + 0.46$$

### Prospective validation of injection protocols integrating IDR_IDEAL_

In the prospective study part that used Eq. 10 to determine the personalized IDR, coronary enhancement of neither group B1 (characterized by an injection rate of 3.5 mL/s) nor group B2 (characterized by an injection rate of 5.0 mL/s) showed a significant correlation with any body metric (*p* > 0.05). All correlation coefficients showed very weak to weak correlations, ranging from 0.019 to 0.27 and 0.016 to 0.20 for group B1 and B2, respectively.

**Fig. 4:**
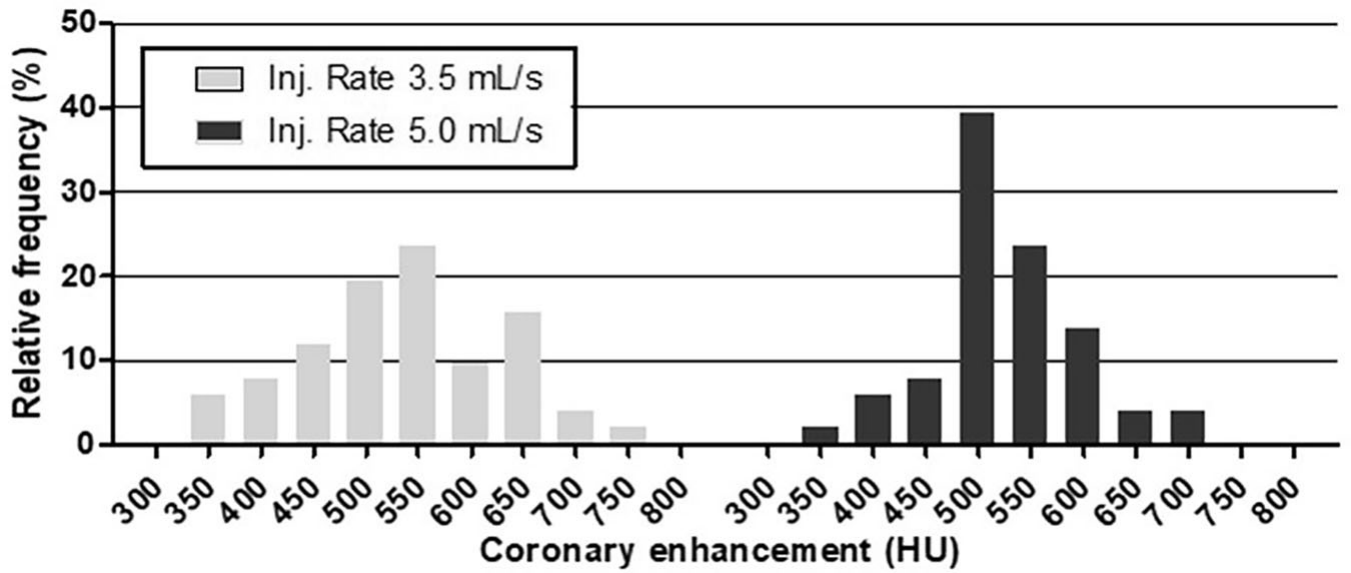
Mean coronary enhancement distributions in both prospective groups

Mean IDR_IDEAL_ was 1.01 ± 0.11 gI/s for group B1 and 1.07 ± 0.14 gI/s for group B2. Mean coronary enhancements were 533 ± 97 HU and 528 ± 68 HU in group B1 and group B2, respectively, and followed in both groups a normal distribution. Mean coronary enhancement between both groups showed no significant difference (*p* = 0.79); however, the variance in HU enhancement in group B2 was significantly lower (*p* = 0.015) compared to group B1. This observation is present in the histograms representing HU distribution in Fig. 4: there is a well-defined HU peak around the 500–550 HU bins in group B2 compared to a wider bell-shaped curve in group B1. Furthermore, only the coronary enhancement in group B2 (mean HU [90% CI]; 528 HU [512–544 HU]) was deemed equivalent with the set target value of 500 ± 50 HU, while this was not the case for group B1 (mean HU [90% CI]; 533 HU [510–556 HU]). Additional information on TID, set IDR, injected ICM volume, heart rate, time to scan, z-axis coverage, scan time, chosen IQ level, radiation dose, SNR and CNR are listed in Table 3.

**Table 3:** Scan timing, injector settings, coronary enhancement, heart rate and radiation dose estimates

	Group B1 Injection rate = 3.5 mL/s	Group B2 Injection rate = 5.0 mL/s	*p*-value
No. of patients	51	51	
Time to scan (s)	30 ± 3	28 ± 3	*p* < 0.01
Z-axis coverage (cm)	20 ± 1	20 ± 1	*p* = 0.79
Scan time (s)	0.3 ± 0.02	0.3 ± 0.02	*p* = 0.79
TID (gI)	17.4 ± 1.9	18.2 ± 2.5	*p* = 0.06
ICM volume (mL)*	50 ± 5	52 ± 7	*p* = 0.06
IDR (gI/s)	1.01 ± 0.11	1.07 ± 0.14	*p* = 0.03
Heart rate (bpm)	60 ± 7	61 ± 8	*p* = 0.40
Mean coronary enhancement (HU)	533 ± 97	528 ± 68	*p* = 0.79
Aorta ascendens (HU)	531 ± 98	529 ± 68	*p* = 0.91
Proximal RCA (HU)	535 ± 98	527 ± 71	*p* = 0.68
IQ level 50 (%)	76	49	*p* < 0.01
IQ level 110 (%)	24	51	*p* < 0.01
CTDI_VOL_ (mGy)	2.7 ± 1.5	3.7 ± 2.0	*p* < 0.01
DLP (mGy*cm)	53 ± 30	72 ± 41	*p* < 0.01
SNR	18 ± 5	19 ± 5	*p* = 0.85
CNR	22 ± 5	23 ± 5	*p* = 0.95

All data are summarized as mean ± standard deviation

*TID* total iodine dose, *ICM* iodinated contrast media, *IDR* iodine delivery rate, *RCA* right coronary artery, *CTDI*_*VOL*_ volume computed tomography dose index, *DLP* dose length product, *SNR* signal-to-noise ratio, *CNR* contrast-to-noise ratio, *IQ* image quality

* Utilizing an ICM with a base concentration of 350 mgI/mL

### Visual grading analysis of coronary enhancement

Overall coronary enhancement was scored excellent by both readers and median [Q1–Q3]) were: 5 [4, 5] vs 5 [4, 5] and 5 [4, 5] vs 5 [5] for group B1 and B2, respectively. For both readers, no significant difference was observed between the two groups (*p* = 0.84 and 0.28). Additionally, per-vessel enhancement scores showed no significant differences between the two groups for both readers (*p* > 0.05). Subjective enhancement scores are listed in Table 4. The objective analysis in terms of SNR and CNR did not show any significant differences either (*p* = 0.85 and 0.95).

**Table 4:** Visual grading analysis of coronary enhancement

	Group B1 Injection rate = 3.5 mL/s		Group B2 Injection rate = 5.0 mL/s	
Overall coronary enhancement	5 [4, 5]	5 [4, 5]	5 [4, 5]	5 [5]
Left main	5 [4, 5]	5 [5]	5 [4, 5]	5 [5]
Proximal RCA	5 [4, 5]	5 [5]	5 [4, 5]	5 [5]
Mid RCA	5 [4, 5]	5 [5]	5 [4, 5]	5 [4, 5]
Distal RCA	5 [4, 5]	4 [3–5]	5 [4, 5]	4 [3–5]
Proximal LAD	5 [4, 5]	5 [5]	5 [4, 5]	5 [5]
Mid LAD	5 [4, 5]	5 [4, 5]	5 [4, 5]	5 [4, 5]
Distal LAD	5 [4, 5]	4 [3–5]	5 [4, 5]	4 [3–5]
Proximal LCx	5 [4, 5]	5 [5]	5 [4, 5]	5 [5]
Mid LCx	5 [4, 5]	5 [3–5]	5 [4, 5]	4 [4, 5]
Distal LCx	5 [4, 5]	4 [2–4]	5 [4, 5]	3 [2–4]

All data are summarized as median [Q1–Q3]

*RCA* right coronary artery, *LAD* left anterior descending artery, *LCx* left circumflex artery

## Discussion

Our study demonstrated that an IDR-based injection protocol with a constant injection rate of 5.0 mL/s resulted in a more precisely targeted coronary enhancement, compared to a lower injection rate of 3.5 mL/s. The injection protocol implemented a personalized IDR, estimated with the calculated FFM_D_ of the patient, using a fixed injection time and, consequently, a calculated contrast agent dilution percentage.

FFM_D_ and LBW were identified as the best candidates for the prediction of the IDR that would achieve the target enhancement, followed by BSA, patient weight and length. Patient age, BMI and WED showed weak correlations. These results are in agreement with findings of previous work [9, 10, 20–22, 28]. We decided to use FFM_D_, which takes into account both the fat-free body composition and patient’s sex, to predict IDR_IDEAL_ (Eq. 10). These parameters seem to be best in representing the volumetric important measures of the cardiac system and vasculature for the application of cCTA.

The integration of IDR in injection protocols is an interesting concept, as it captures the impact of both injection rate and iodine concentration on coronary enhancement. Various phantom and clinical studies have already shown the utility of this parameter, obtaining similar enhancement levels when using different contrast media concentrations [32, 33, 41, 42]. In agreement with these previous studies, we found that the mean coronary enhancement in both prospective study groups was not significantly different. Additionally, our results showed that coronary enhancement and the body metrics were no longer significantly correlated in the prospective groups, proving that our proposed formula for personalized IDR (Eq. 10) successfully takes into account body habitus. This is what was aimed for.

With the introduction of PCCT, the need for high iodine concentration injections is history as VMIs of lower energies are standard-of-care and could be calculated for every patient. As determined in this study, an IDR ranging from 0.85 to 1.30 gI/s is not exceptional. Similar IDR values were also obtained by Emrich et al [35]. Implementing these low IDR values, for example, with a fixed iodine concentration of 350 mgI/mL, would result in injection rates ranging from 2.4 to 3.7 mL/s. It should be noted that the implementation of these lower injection rates should be well considered, as our data indicate that the injection rate has an impact on coronary enhancement distribution throughout the patient population. In this study, the prospective group, characterized by 3.5 mL/s injection rate, showed a significantly higher variance in coronary enhancement, compared to the group characterized by 5.0 mL/s injection rate. We hypothesize that these differences can be (in part) explained by (1) the total volume and (2) the velocity of the injected bolus. In our study, the injected bolus volumes were fixed per injection rate, with however saline replacing iodine to tune the IDR. Total volume of the injected bolus in the 3.5 mL/s group was 60 mL, compared to 85 mL in the 5 mL/s group. We assume that injecting a higher volume using a higher injection rate allows less dispersion of the contrast bolus in the blood, compared to a smaller and more slowly injected bolus and is therefore less susceptible to underlying patient-specific factors beyond body habitus.

This study is limited in that it is a mono-centric study with only a limited number of patients included. Second, it should be noted that the calculated IDR_IDEAL_ was selected as an approximation for the ideal coronary enhancement. We calculated IDR_IDEAL_ in the retrospective group based on the assumption that coronary enhancement and iodine concentration relate linearly. Furthermore, the coronary enhancement was only measured in the ascending aorta and proximal right coronary artery, which was defined as an indication for total coronary enhancement. Additionally, in the retrospective cohort, injection rate varied from 3.0 to 4.0 mL/s. As discussed in this study, the HU value distribution throughout the included patient population could be affected, and therefore, coronary enhancement does not depend only on the calculated IDR. Third, cardiac output was not implemented in this study, and therefore, the impact was not assessed.

Notwithstanding the limitations, these findings show the advantage of maintaining a sufficiently high injection rate in injection protocols for cCTA, even if low IDR rates can be used. Future work could include the potential of VMI-based injection protocols, ultimately tailoring contrast injections both to the patient and clinical task [43, 44]. If a higher or lower HU would be aimed for, the dilution rates would have to be increased or lowered linearly, at least in a first approximation. For example, a target HU of 425 in 55 keV reconstructed images would already allow a further TID reduction of 15%.

In conclusion, this study proposed and validated a personalized formula for an IDR-based injection protocol in cCTA. The fat-free mass body metric was derived as the best candidate for the prediction of ideal IDR. Consequently, the impact of injection rate was assessed and showed that a higher injection rate resulted in more precisely targeted coronary enhancement throughout the patient population.

## Abbreviations

BMI: Body mass index
BSA: Body surface area
CAD: Coronary artery disease
cCTA: Coronary computed tomography angiography
CNR: Contrast-to-noise ratio
FFM: Fat-free mass
ICM: Iodinated contrast media
IDR: Iodine delivery rate
LBW: Lean body weight
PCCT: Photon-counting computed tomography
ROI: Region of interest
SNR: Signal-to-noise ratio
TID: Total iodine dose
VMI: Virtual mono-energetic image
WED: Water equivalent diameter
